# Revised criteria for light chain MGUS enhance diagnostic accuracy and risk stratification

**DOI:** 10.1038/s41408-026-01478-y

**Published:** 2026-03-30

**Authors:** Lærke Sloth Andersen, Cecilie Velsø Mæng, Sæmundur Rögnvaldsson, Thórir Einarsson Long, Christian Brieghel, Emil Hermansen, Morten Nørgaard Andersen, Kirsten Grønbæk, Carsten Utoft Niemann, Sigurður Yngvi Kristinsson, Sigrún Thorsteinsdóttir

**Affiliations:** 1 Department of Hematology, Rigshospitalet, Copenhagen University Hospital, Copenhagen, Denmark; 2 Biotech Research and Innovation Center (BRIC), University of Copenhagen, Copenhagen, Denmark; 3 Faculty of Medicine, University of Iceland, Reykjavik, Iceland; 4 Department of Hematology and Coagulation, Sahlgrenska University Hospital, Gothenburg, Sweden; 5 Department of Nephrology, Skåne University Hospital, Lund, Sweden; 6 Department of Clinical Sciences, Lund University, Lund, Sweden; 7 Danish Cancer Institute, Copenhagen, Denmark; 8 Department of Hematology, Aarhus University Hospital, Aarhus, Denmark; 9 Department of Clinical Medicine, Aarhus University, Aarhus, Denmark; 10 Department of Molecular Medicine, Aarhus University Hospital, Aarhus, Denmark; 11 Department of Clinical Medicine, Faculty of Health and Medical Sciences, University of Copenhagen, Copenhagen, Denmark; 12 Landspitali University Hospital, Reykjavik, Iceland

**Keywords:** Myeloma, Epidemiology, Cancer epidemiology, Myeloma

## Abstract

Light chain monoclonal gammopathy of undetermined significance (LC-MGUS) is an asymptomatic precursor of multiple myeloma (MM) and related disorders defined by abnormal free light chain (FLC) testing. Recently, revised FLC reference intervals were proposed by the iStopMM study group. We evaluated their performance in a large population-based cohort. MGUS cases were identified from the Danish Lymphoid Cancer Research (DALY-CARE) data resource, including only individuals with Freelite FLC-measurements. Cases were classified by original and revised LC-MGUS criteria, hereby identifying cases only fulfilling original but not revised criteria (reclassified as normal). Outcomes were progression to MM or related disorders. Cumulative incidence of progression with death as competing risk was examined using Aalen-Johansen estimation. Annual progression risk was assessed using a person-years approach. In total, 360 individuals classified by original criteria, 215 by revised, while 150 (40%) were reclassified as normal. In the revised group, there were 21 (9.8%) progressions; 11 (5.1%) to MM and seven (3.3%) to AL amyloidosis. Only two individuals (1.3%) progressed in the reclassified group, none to MM or AL amyloidosis. For revised LC-MGUS, the 2- and 5-year cumulative incidence of progression was 5.8% and 8.9%, respectively, with an annual progression rate of 3%. This study validates the performance of the iStopMM revised LC-MGUS criteria in a clinical cohort. Applying the revised criteria reduced LC-MGUS diagnoses by 40% without missing cases progressing to MM or AL amyloidosis. These findings confirm the accuracy of the revised criteria and support their implementation to improve diagnostics and ensure follow-up of individuals truly at risk.

## Introduction

Monoclonal gammopathy of undetermined significance (MGUS) is an asymptomatic precursor condition of multiple myeloma (MM), and other lymphoproliferative diseases (LPD), with a prevalence of approximately 4–5% in individuals over 50 years of age, and an average annual risk of progression of 1% [1, 2]. MGUS encompasses several biological subtypes, including light chain MGUS (LC-MGUS), which is defined by an abnormal serum free light chain (FLC) ratio, an elevated level of the involved light chain, absence of a heavy chain monoclonal protein, and no evidence of end-organ damage attributable to the gammopathy. Previous studies have estimated a prevalence of LC-MGUS of 0.8% among individuals over 50 years old with an annual risk of progression to malignant disease of approximately 0.3% [3].

Current guidelines recommend follow-up and more comprehensive evaluation for individuals with high-risk MGUS, as defined by the Mayo Clinic risk stratification model, which incorporates established risk factors reflecting the plasma cell burden: M-protein level ( > 1.5 g/dL), immunoglobulin (Ig) type (non-IgG), and abnormal FLC-ratio [4]. In LC-MGUS, where M-protein is absent, and classification relies on an abnormal FLC ratio, other factors must be considered when assessing progression risk. Some clinical guidelines recommend using an involved/uninvolved FLC-ratio cut off at 8 or 10, where higher ratios should warrant further evaluation, such as bone marrow (BM) examination and radiological imaging [5–8]. In addition to high FLC-ratio, immunoparesis has previously been found to be a risk factor of progression in LC-MGUS [7, 9].

As the FLC ratio is a key diagnostic and prognostic marker in LC-MGUS, consequently the accuracy of FLC measurements, and their interpretation, is crucial. The current FLC reference intervals derive from a relatively small study of 282 individuals and recent findings have questioned the validity and representability within the general population [10–12]. Recently published data from the Iceland Screens, Treats, or Prevents Multiple Myeloma (iStopMM) study addressed this issue by proposing FLC reference intervals adjusted for age and kidney function, based on their screened cohort of 75,422 individuals [13, 14]. Application of these revised intervals, and thus revised diagnostic criteria for LC-MGUS, in the iStopMM cohort reduced the prevalence of LC-MGUS by 82% without compromising prognostic performance and has later been validated externally [9, 15, 16]. In addition, we recently validated the revised reference intervals for MGUS with an intact M-protein demonstrating improved prognostic accuracy by correctly identifying high-risk individuals while confirming that those reclassified to normal FLC values had no increased risk of progression [17].

Previous studies have shown that patients with a known prior diagnosis of MGUS who subsequently develop MM have improved outcomes, likely owing to closer monitoring and earlier initiation of therapy [18, 19]. But improving diagnostic precision and risk stratification in LC-MGUS is not only of clinical importance - it also impacts healthcare economics by reducing unnecessary evaluation and follow-up in individuals with falsely abnormal FLC results and relieving strain on specialist services.

Thus, we aimed to evaluate the performance of the iStopMM revised LC-MGUS diagnostic criteria and explore risk factors associated with progression in a clinical cohort. By reassessing the natural history of LC-MGUS under the new diagnostic framework, we aimed to improve the precision of risk stratification, reduce unnecessary evaluation, and ensure that follow-up is reserved for those at genuine risk.

## Methods

### Data collection

This nationwide retrospective cohort study utilized data from the Danish Lymphoid Cancer Research (DALY-CARE) data resource, which includes all Danish adults diagnosed with a lymphoproliferative cancer or precursor condition between 2002 and 2024 [20]. Records were linked using the Danish civil registration (CPR) number enabling integration of information from multiple nationwide sources, including registries maintained by the Danish Health Data Authority and the Danish Healthcare Quality Institute (SundK) as well as regional electronic health records (EHR) from the Capital Region and Region Zealand covering half of the Danish population.

### LC-MGUS cohort

Since LC-MGUS does not have a specific diagnostic code, identification of LC-MGUS cases was based on MGUS International Classification of Diseases, 10th Revision (ICD-10) diagnosis codes as recorded in the Danish National Patient Registry (DNPR), the Danish Myeloma Database (DaMyDa), the National Pathology Data Bank, or MGUS diagnosis codes registered in EHR within the DALY-CARE platform. There is no population-level screening for MGUS in Denmark; instead, testing is initiated when clinical findings raise suspicion of an underlying hematologic disorder, in accordance with national guidelines from the Danish Health Authority and the Danish Myeloma Study Group. Laboratory results were extracted using Nomenclature, Properties and Units (NPU) coding system (Supplementary Table 1). Individuals with evidence of a heavy chain M-protein on serum protein electrophoresis (SPEP) or immunofixation electrophoresis (IFE), either prior to or within one year following the MGUS diagnosis date, were excluded. Individuals who did not have a detectable M-protein at the time of MGUS diagnosis but were found to have an M-protein more than one year later were included in the study, as this was considered indicative of disease progression rather than reflective of their initial disease state.

The analysis of serum FLC as part of the diagnostic work-up of monoclonal gammopathies was introduced in Denmark in 2007. Thus, individuals diagnosed with MGUS before 2007 were excluded in this study along with individuals without relevant laboratory results. Only cases with FLC measurement within 180 days of the MGUS diagnosis and creatinine measurements within 30 days of the FLC measurement were included. Furthermore, only FLC results from laboratories using the FreeLite assay were included, because the revised iStopMM reference intervals were developed using this assay. We excluded individuals with a concomitant diagnosis of MM, B-cell non-Hodgkin lymphoma (NHL) (including chronic lymphoid leukemia and Waldenström’s macroglobulinemia) or amyloid light chain (AL) amyloidosis prior to or within 90 days of MGUS diagnosis. MM, NHL and AL amyloidosis diagnoses were identified in DALY-CARE using ICD-10 codes (Supplementary Table 2).

**Fig. 1:**
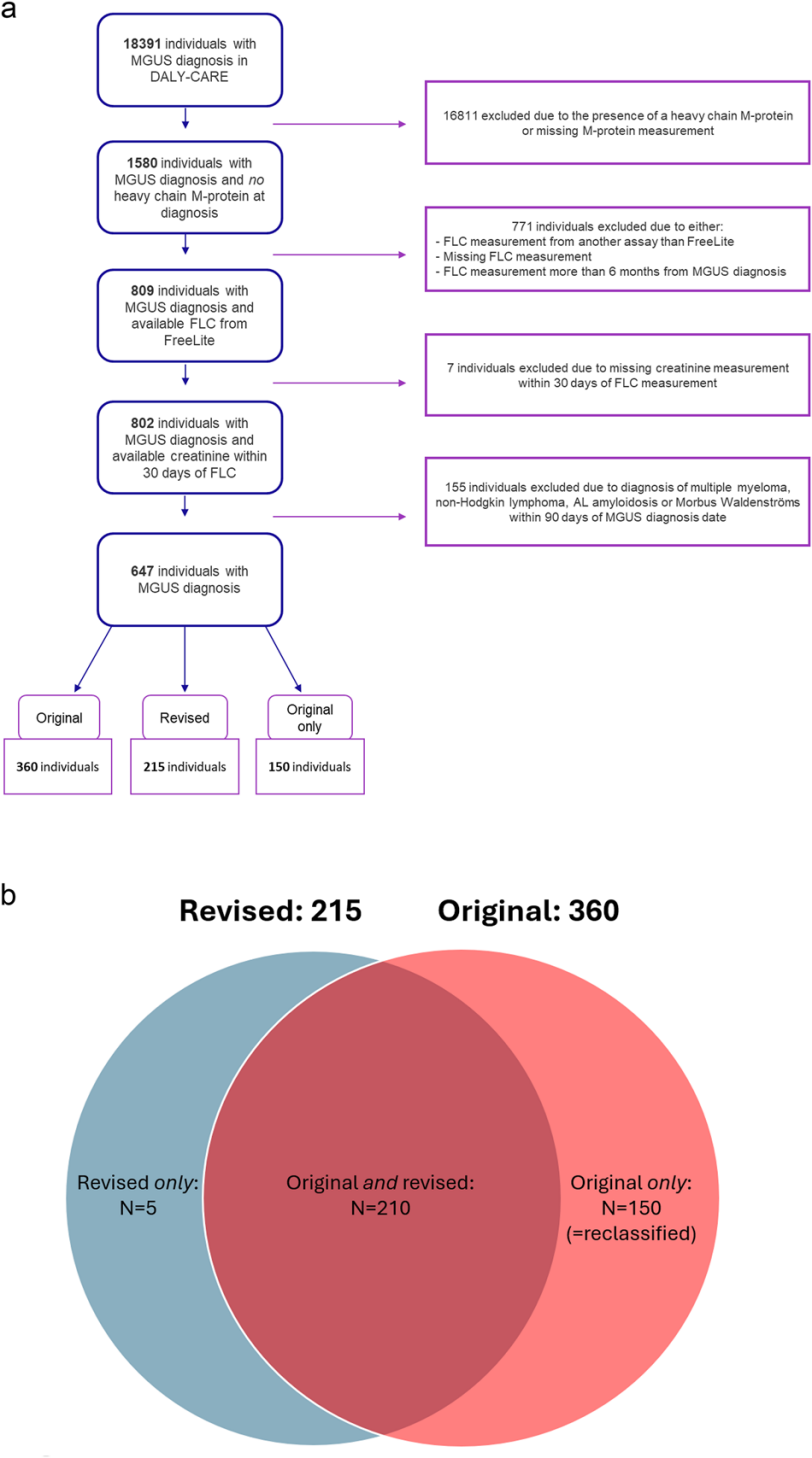
Identification and distribution of study cohort. **a** Flow diagram illustrating the identification of the study cohort. The diagram shows the total number of individuals in the Danish Lymphoid Cancer Research (DALY-CARE) data resource, with excluded individuals in pink and included individuals in blue. **b** Venn diagram showing the overlap between individuals meeting the original (red) and revised (blue) LC-MGUS diagnostic criteria, as well as individuals meeting only the revised or only the original criteria in the Danish Lymphoid Cancer Research (DALY-CARE) cohort. MGUS monoclonal gammopathy of undetermined significance, M-protein monoclonal protein, FLC free light chain, AL amyloid light chain. LC-MGUS light chain monoclonal gammopathy of undetermined significance.

For analysis, participants were categorized based on the LC-MGUS criteria they fulfilled (supplementary Table 3): *original LC-MGUS*, defined by an abnormal FLC ratio with elevation of the involved FLC according to the original reference intervals; *revised LC-MGUS*, defined by the same criteria but using the revised iStopMM reference intervals; and lastly cases meeting *only* the original but *not* the revised criteria (reclassified as normal according to the iStopMM definition), hereafter referred to as the *reclassified* group [10, 13]. The date of MGUS diagnosis was defined as the date of the first FLC measurement closest to the MGUS diagnosis date. The cohort identification and inclusion process are summarized in Fig. 1a.

### Outcomes

The primary outcomes were progression to any LPD, including both MM, B-cell NHL, and AL amyloidosis, identified by ICD-10 codes in DALY-CARE (listed in Supplementary Table 2). A sensitivity analysis was performed with progression to only MM or AL amyloidosis as outcome. Individuals were followed from the set MGUS diagnosis date until progression, death, or end of follow-up. Follow-up was terminated on November 30, 2024, to ensure data availability from all used sources on vital status, and complete follow-up. Thus, individuals diagnosed after this date were excluded from progression analyses.

To describe the clinical characteristics of individuals who progressed to MM, with particular focus on the type of organ involvement present at the time of progression, registration in DaMyDa and laboratory data was reviewed. Finaly, we performed an exploratory analysis to identify possible risk factors of progression in the revised LC-MGUS group, including FLC-ratio, kappa vs. lambda isotype and immunoparesis, defined as suppression of at least one Ig.

### Statistical analysis

Descriptive characteristics of the LC-MGUS cohort were presented for the three groups: original, revised, and reclassified. Individuals that met the diagnostic criteria for both original and revised MGUS were included in both groups. All analyses were performed in R using the gtsummary and tidyverse packages [21, 22]. The median follow-up was assessed using the reversed Kaplan-Meier method.

The estimated cumulative incidence of progression to MM, B-cell NHL, or AL amyloidosis was calculated and visualized graphically using the Aalen-Johansson estimator, considering death as a competing risk. Gray’s test was used to evaluate differences in cumulative incidence. The standard Kaplan-Meier estimator was used to assess the cumulative incidence of progression without death as competing risk and progression rates were calculated as events per total person-time, expressed per 100 person-years, providing an average hazard rate, that approximates the annual risk of progression. This approach was selected to ensure comparability with prior MGUS studies [3, 23]. Hazard ratios (HR) were calculated using Cox regression, censoring at death or end of follow-up. HRs were presented crude and adjusted for age and sex. Schoenfeld residuals were used to assess the proportional hazards assumption, and martingale residuals were examined to assess the linearity assumption for continuous covariates. All analyses were performed in R using the prodlim and survival packages [24, 25].

## Results

### The study cohort—baseline characteristics

A total of 360 individuals classified as LC-MGUS by the original criteria, 215 by the revised criteria (of which 210 met both original and revised criteria, and 5 met only the revised), and 150 met *only* the original criteria (the reclassified group), as shown in Fig. 1b. Implementation of the revised criteria thus resulted in the reclassification of 150 individuals, who no longer met the diagnostic criteria for LC-MGUS. The five individuals who only fulfilled the revised criteria had thus received an MGUS diagnosis without fulfilling the original criteria (all had either increased kappa or lambda light chain but an FLC-ratio within the original reference values). Consequently, the absolute number of LC-MGUS diagnoses was reduced by approximately 40%.

The median age was 72 years in all three groups. The sex distribution differed among the revised and reclassified groups with 61% men in the revised group compared to 55% in the reclassified group. Kappa LC-MGUS accounted for 64% of cases in the revised group and 99% in the reclassified group. Among the five individuals who only met the revised criteria, four had lambda LC-MGUS. Lastly, more individuals in the revised group had immunoparesis with 79 (37%) individuals compared to 20 (14%) individuals in the reclassified group (Table 1). Baseline characteristics are presented in Table 1.

**Table 1:** Descriptive data of individuals with LC-MGUS in DALYCARE.

	LC-MGUS revised	LC-MGUS original	Reclassified individuals
Characteristic	*N* = 215	*N* = 360	*N* = 150
Age (years), Median (IQR)	72 (64 – 80)	72 (66 – 78)	72 (66 – 77)
Sex, n (%)			
F	83 (39)	150 (42)	68 (45)
M	132 (61)	210 (58)	82 (55)
Lambda (mg/L), Median (IQR)	26 (13–140)	18 (13–47)	16 (12–23)
Kappa (mg/L), Median (IQR)	89 (24–207)	47 (26–139)	35 (27–47)
FLC-ratio*, Median (IQR)	9.75 (5.37–20.00)	4.66 (2.28–13.00)	2.16 (1.89–2.60)
eGFR (mL/min/1.73m^2^), n (%)			
>=60	168 (78)	295 (82)	131 (87)
0–14	4 (1.9)	7 (1.9)	3 (2.0)
15–29	7 (3.3)	17 (4.7)	10 (6.7)
30–44	17 (7.9)	21 (5.8)	4 (2.7)
45–59	19 (8.8)	20 (5.6)	2 (1.3)
Lambda LC MGUS, n (%)	77 (36)	75 (21)	2 (1.3)
Kappa LC MGUS, n (%)	138 (64)	285 (79)	148 (98.7)
Immunoparesis in at least 1 Ig, n (%)	79 (37)	98 (27)	20 (14)
Immunoparesis in >1 Ig, n (%)	23 (11)	25 (7.0)	2 (1.4)
Events during follow-up, n (%)			
Deaths within follow-up, n (%)	58 (27)	108 (30)	52 (35)
Multiple myeloma, n (%)	11 (5.1)	9 (2.5)	0 (0)
Lymphoma, n (%)	2 (0.9)	4 (1.1)	2 (1.3)
AL amyloidosis, n (%)	7 (3.3)	6 (1.7)	0 (0)
Morbus Waldenström, n (%)	1 (0.5)	1 (0.3)	0 (0)

^*^Involved/uninvolved FLC-ratio.

*MGUS* monoclonal gammopathy of undetermined significance, *IQR* interquartile range, *FLC* free light chain, *eGFR* estimated glomerular filtration rate, *Ig* immunoglobulin, *AL* amyloid light chain.

A total of 26 individuals were excluded from the progression analyses due to lack of follow-up data on vital status. Calculations of the cumulative incidence of progression therefore included 334 individuals in the original group, 191 individuals in the revised group, and 148 individuals in the reclassified group (Supplementary Table 4), yielding a total of 339 unique individuals with complete data (revised + reclassified). The median follow-up time was 3.73 years (IQR 1.48–6.91). In the revised LC-MGUS group, for those with complete follow-up data, there were 21 progressions of which 11 (5.8%) progressed to MM, seven (3.7%) to AL amyloidosis, and three to NHL (1.5%) (including one to Waldenström’s macroglobulinemia). In the reclassified group, only two (1.4%) individuals progressed, both to NHL. A total of 58 (30%) individuals in the revised group and 52 (35%) individuals in the reclassified group, died prior to progression to any LPD.

### Characteristics of progressors

Of the 11 individuals who progressed to MM, 5 could be verified to fulfill a CRAB criterion at time of diagnosis and an additional patient fulfilled SLiM CRAB (FLC-ratio>100). Two patients likely had SMM based on review of medical records. The three remaining patients did not have sufficient data to estimate CRAB at progression.

### Risk of progression

#### Progression to any LPD

**Fig. 2:**
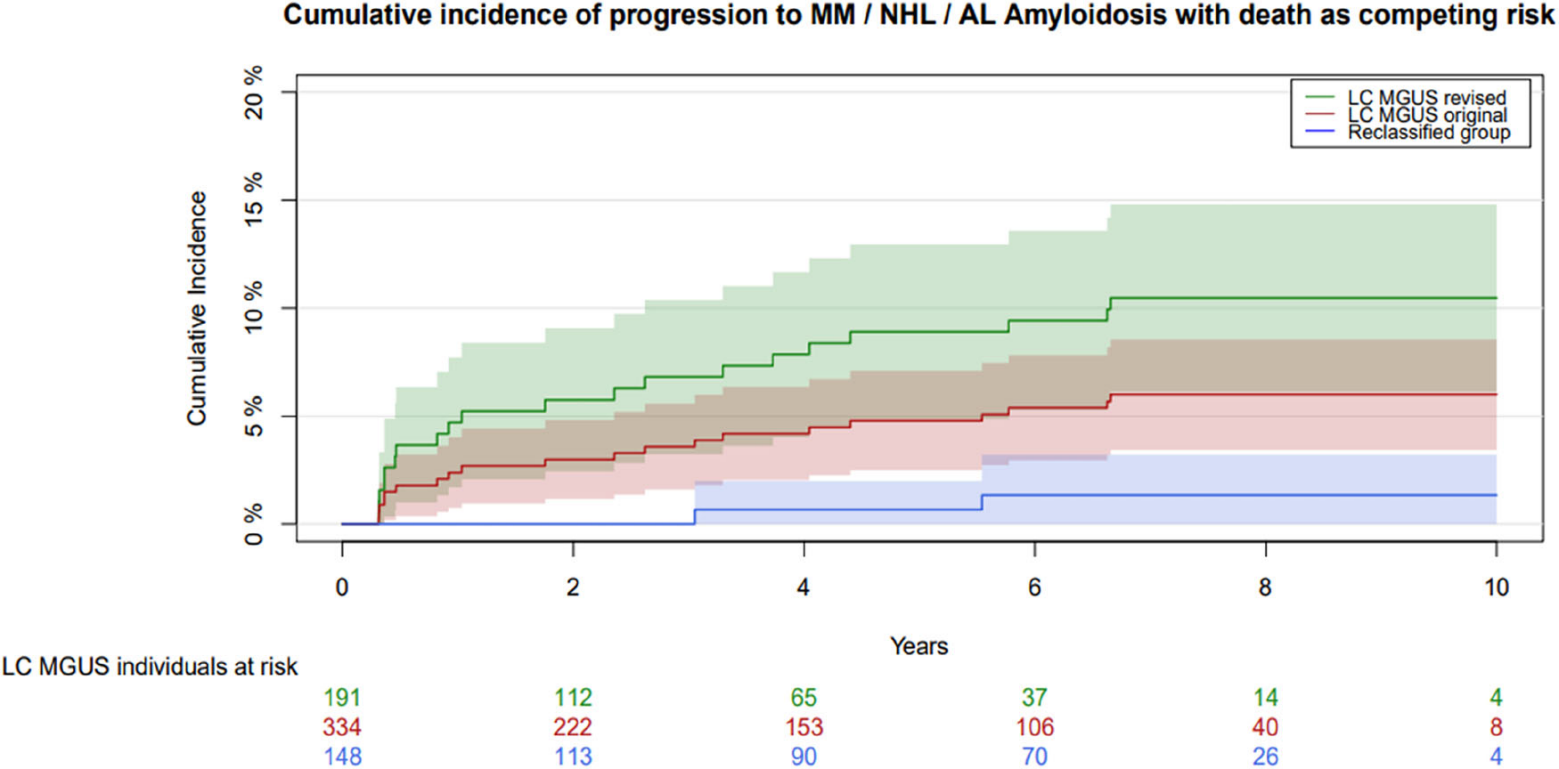
Cumulative incidence curves showing the risk of progression to any LPD in individuals with LC-MGUS, using Aalen-Johansson estimation accounting for death as competing risk. The three curves represent the revised LC-MGUS group (green), the original LC-MGUS group (red) and the reclassified group (blue). LPD lymphoproliferative disease, LC MGUS light chain monoclonal gammopathy of undetermined significance, MM multiple myeloma, NHL non-Hodgkin’s lymphoma (including Morbus Waldenströms), AL amyloid light chain.

Accounting for death as competing risk, the cumulative incidence of progression to any LPD was 5.8% (95% confidence interval (CI): 2.5–9.1) at 2 years, and 8.9% (95% CI: 4.9–12.9) at 5-years, for the revised LC-MGUS group (Fig. 2). Not accounting for death as competing risk, the cumulative incidence of progression was 6.6% (95% CI: 2.7–10.3) and 13.7% (95% CI: 6.8–20.0) at 2 and 5 years, respectively.

For the reclassified group, the 2- and 5-year cumulative incidence of progression to any LPD was 0.0% and 0.7% (95% CI: 0.0–0.2), respectively (Fig. 2), accounting for death as competing risk, compared to 0.0% and 1.0% (95% CI: 0.0–3.0) not accounting for death as competing risk.

The incidence rate of progression to any LPD was significantly lower in the reclassified group with 2.6 events per 1000 person-years, compared to an incidence rate of 30.9 events per 1000 person-years in the revised LC-MGUS group (*p* = 0.0014) corresponding to a progression rate of 0.26% and 3.1% per year, respectively. Both progressions in the reclassified group were to NHL and not to MM or AL amyloidosis.

When only progression to MM or AL amyloidosis was considered as outcome, the cumulative incidence results were similar (Supplementary Fig. 1).

There was no difference in cumulative incidence of death prior to progression to LPD between the revised, original, and reclassified groups (Gray’s test: *p* = 0.82), visualized in Supplementary Fig. 2.

### Risk factors of progression

#### FLC-ratio

**Fig. 3:**
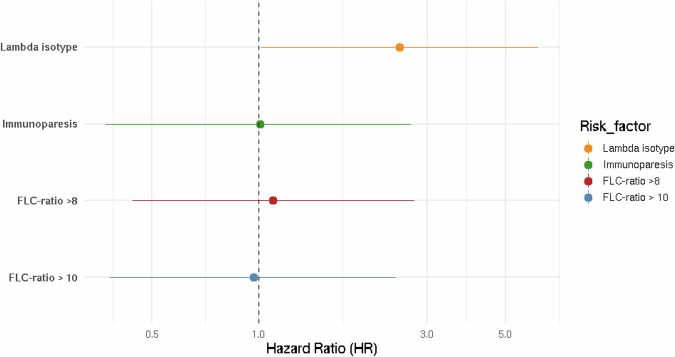
Forest plot illustrating associations between risk factors at LC-MGUS diagnosis and risk of progression to lymphoproliferative disorder (LPD). Hazard ratios (HR) are adjusted for sex and age. Full model estimates are provided in Supplementary Table 5. LC MGUS light chain monoclonal gammopathy of undetermined significance, FLC free light chain. Immunoparesis defined as suppression of at least one immunoglobulin.

Individuals with an FLC-ratio >10 did not have a significantly higher risk of progression to LPD compared to individuals with an FLC-ratio <10 (HR 0.97, 95% CI: 0.38–2.45) (Fig. 3). Similarly, using an FLC-ratio cut off at 8 revealed no statistically significant difference in progression risk in individuals with an FLC-ratio >8 compared to individuals with an FLC-ratio <8 (HR 1.10, 95% CI: 0.44–2.77). Notably only 2 of the 7 individuals who progressed to AL amyloidosis had a baseline FLC-ratio >10 and 4 had a baseline FLC-ratio >8. However, when considering progression to MM only, the estimated risk of progression was higher among individuals with an FLC-ratio >10 (HR 2.06, 95% CI: 0.58–7.29), although this was not statistically significant.

#### FLC isotype

Individuals with lambda LC-MGUS had a significantly higher risk of progression to LPD compared to those with kappa LC-MGUS (HR 2.51, 95% CI: 1.01–6.20). When progression to MM alone was considered, the estimate still indicated a higher risk associated with having lambda LC-MGUS (HR 2.12, 95% CI 0.61–7.27).

#### Immunoparesis

The presence of immunoparesis was not associated with a statistically significant difference in progression risk (HR 1.01, 95% CI: 0.37–2.70) to any progression, nor when progression to MM only was considered (HR 0.41, 95% CI: 0.08–2.56).

## Discussion

In this population-based cohort study, implementation of the iStopMM revised FLC reference intervals reduced the number of LC-MGUS diagnoses by 40%. None of the reclassified individuals progressed to MM or AL amyloidosis, confirming that the revised criteria safely eliminate false-positive cases. Among those meeting the revised criteria, LC-MGUS carried a 3% annual risk of progression, which is higher than previously assumed. This indicates that the revised criteria both improve diagnostic precision and sharpen prognostic accuracy allowing follow-up to focus on individuals truly at risk.

Our finding of a 40% reduction in LC-MGUS diagnoses using the revised reference intervals is lower than in previously published data [9, 15]. This likely reflects that our study is based on a clinical cohort, with a selected patient population that have received a diagnostic code of MGUS, thus demonstrating how the use of the revised reference intervals can help reduce incorrect diagnoses in clinical practice relieving the possible psychological burden affecting individuals going through a further diagnostic process and receiving a premalignant diagnosis. Optimization of LC-MGUS diagnostics may decrease the frequency of imaging studies, BM examinations, and physician consultations. Such improvements could yield economic benefits across diverse healthcare systems by promoting more efficient resource utilization, reducing demands on specialist care, and minimizing the burden on patients.

When applying the revised criteria, we observed that 99% of those that no longer met the diagnostic criteria for LC-MGUS had kappa LC-MGUS isotype. This is in line with prior studies that have documented a drift toward higher kappa/lambda ratios over time in the absence of monoclonal disease, possibly due to small calibration changes in the Freelite assay. Notably, specificity for monoclonal kappa elevation has been shown to be substantially reduced within the FLC-ratio range of 1.65–3.0, which is also reflected among the reclassified individuals in this study (median FLC-ratio 2.16; IQR 1.89–2.60) [11, 12, 26]. Although FLC values are influenced by age and renal dysfunction, which tends to elevate kappa disproportionately, neither renal impairment nor age differed between the reclassified and revised groups (Table 1). In contrast, four out of the five individuals who met only the revised criteria had lambda LC-MGUS. Together, these findings suggest that the original reference intervals contribute to substantial overdiagnosis of kappa LC-MGUS and an underdiagnosis of lambda LC-MGUS, whereas using the revised reference intervals can reduce false-negative lambda LC-MGUS diagnoses and false-positive kappa LC-MGUS cases. This was further supported by the fact that two patients that only met the revised lambda LC-MGUS criteria, progressed to MM during follow-up. This underscores the diagnostic accuracy of the revised criteria, highlighting their potential to improve early detection of clinically relevant disease.

Importantly, we found that none of the reclassified individuals progressed to MM or AL amyloidosis. The two reclassified individuals who did progress were diagnosed with NHL, and the incidence of 2.6 events per 1000 person-years for the reclassified group is slightly higher than the NHL incidence in the background population of Denmark above 50 years [27]. However, the risk of NHL is increased in the elderly and males, which are both overrepresented in our cohort compared to the general population. Thus, the two observed NHL events may not be related to their previous LC-MGUS diagnosis. Nevertheless, our results demonstrate that the reclassified individuals are not at an elevated risk of plasma cell malignancy, which is essential, as it supports the safety of the implementation of the revised criteria in clinical practice.

We examined the risk of progression in the revised LC-MGUS group and found that the LC-MGUS diagnosis carried a higher risk of progression to LPD than previously estimated, approximately 3% per year using a events/person-time approach, not accounting for death as competing risk. By comparison, using the 5-year cumulative incidence of progression with death as competing event, yields an annual progression risk of approximately 1.8% although this estimate is associated with some uncertainty as the progression over time was not completely linear. Regardless of the method used, the annual risk of malignant progression appears higher than the initially reported rate of 0.3% per year [3], aligning more closely with the progression risk observed in conventional MGUS, as is to be expected when removing a substantial number of false positives from the risk set [4]. However, although it has been shown that progression risk for MGUS in general does not differ between screened vs. clinical cohorts [28], it is important to keep in mind that our findings are based on a LC-MGUS cohort, with individuals that are tested in a clinical setting and have received an MGUS diagnosis, rather than a screened cohort. Furthermore, our cohort does not capture all individuals with abnormal FLC testing in Denmark, as not all are referred to hematology services and therefore may not receive an ICD-10–coded MGUS diagnosis, even if they meet diagnostic criteria. This may be particularly true for individuals with only mildly abnormal FLC results which might falsely increase our progression estimate. Finally, because MM and SMM are coded identically in ICD-10, a degree of outcome misclassification cannot be excluded and may contribute to an apparent excess of MM diagnoses. While these factors may influence the observed risk estimate, our results indicate that the progression risk in revised LC-MGUS is higher than previously assumed, warranting further investigation and confirmation in future studies.

When examining the predictive value of an FLC-ratio cut off at 10 or 8 we found that this was a suboptimal stratification of our cohort regarding progression, especially failing to capture the individuals at risk of AL amyloidosis. Lambda LC-MGUS appeared to be associated with higher risk of progression to any LPD, as well as to MM alone. Contrary to previous studies, immunoparesis at diagnosis did not significantly affect progression risk compared to normal Ig levels [7]. However, the lack of significance could be due to a limited study population, and these observations underscore the need for more nuanced risk stratification models for LC-MGUS. In line with that, the iStopMM study group have designed a model that predicts which individuals presumed to have MGUS have ≥10% BM plasma cells, consistent with SMM or MM, which also applies for individuals with LC disease. This prediction model aids clinical decision-making by identifying patients who may safely defer a BM biopsy and those who warrant earlier evaluation [29].

The strengths of this study include the large population-based dataset with data from several nationwide registries. The DALY-CARE integrates information on diagnoses, progressions, laboratory results, and vital status from multiple data resources, allowing acquisition of data also on individuals who are not in clinical follow-up, ensuring a more nuanced study population [20]. However, there are some limitations. The study cohort is a clinical population thereby introducing possible selection bias, which could contribute to an overestimation of progression risk, but gives a good indication of applying the revised reference intervals for FLC in a clinical setting. Because this study is retrospective and relies on prior registrations, it cannot be ruled out that some recorded MM progressions actually represent SMM, as both conditions are coded under the same ICD-10 classification. This may have contributed to a potential overestimation of MM progression events. Furthermore, the individuals with a MGUS diagnosis might be more morbid compared to individuals with unrecognized MGUS, as MGUS is typically discovered as an incidental finding during diagnostic work-up for other conditions. FLC measurements from laboratories using other assays than the Freelite assay were excluded, affecting the generalizability of this study. Finally, we do not have information on ethnicity, however, the iStopMM reference intervals have been validated in ethnically diverse populations [16, 30].

In conclusion, this study validates the performance of the iStopMM revised criteria of LC-MGUS in a clinical population-based cohort by demonstrating its utility in the diagnostics of LC-MGUS as well as enhancing prognostic accuracy, improving care for individuals with LC-MGUS. Implementing the iStopMM revised reference intervals reduces LC-MGUS diagnoses by 40% by removing false-positive LC-MGUS cases. This consequently allows for a more precise identification of individuals with a true monoclonal precursor condition, who appear to have a higher risk of progression than previously assumed. Thereby, this study shows that the revised criteria are both effective and safe to implement and allows health care systems to focus follow-up on those truly at risk. Accordingly, these results provide strong support for the recommendation to implement the revised iStopMM criteria for LC-MGUS in clinical practice.

## Supplementary information

Supplemental material

## Data Availability

The data used in this study are not publicly available to protect the privacy of individuals within the study cohort. However, access can be granted through the Danish Lymphoid Cancer Research (DALYCARE) data resource upon obtaining the required approvals. The R scripts containing the statistical analysis codes are available from the corresponding author upon request.
