# Supplementary material for: Revised criteria for light chain MGUS enhance diagnostic accuracy and risk stratification
Source: Blood Cancer J. 2026 Mar 30;16(1):50. doi: 10.1038/s41408-026-01478-y (PMC13039883; doi:10.1038/s41408-026-01478-y)
Supplement: Supplementary file 1 — Supplemental material [file 41408_2026_1478_MOESM1_ESM.pdf]

Supplementary material

Supplementary Table 1: NPU codes used in the study

Laboratory result	NPU code
Kappa chain free	"NPU19606"
Lambda chain free	"NPU19607",
Kappa chain monoclonal	“NPU28644”
Lambda chain monoclonal	“NPU28645”
Monoclonal IgG	"NPU28638", "NPU28639", "NPU28625", “NPU19807”, “NPU19809”
Monoclonal IgA	"NPU28634", "NPU28635", "NPU28623", “NPU19791”, “NPU19793”
Monoclonal IgM	"NPU28640", "NPU28641", "NPU28926", “NPU19821”, “NPU19823”
Monoclonal IgD	"NPU28636", "NPU28637", "NPU28924"
Monoclonal IgE	"NPU28642", "NPU28643", "NPU28927"
Monoclonal M-protein, unspecified	“NPU17675”, “NPU19844”, “NPU02642”, “NPU19846”
Creatinine	"NPU04998", "NPU18016"

Supplementary Table 2: ICD-10 codes used in the study

Diagnosis	ICD10 codes
MGUS	“DD472”, “DD472A”, “DD472B”
Multiple Myeloma	“DC900”, “DD901”, “DC902”, “DC903”
Non-Hodgkin’s B-cell lymphoma incl. Chronic Lymphoid Leukemia and Morbus Waldenström’s	"DC82X", "DC83X", "DC85X", “DC914”, “DC911”, “DC884”, “DC880”
AL amyloidosis	“DE858A”

Supplementary Table 3: Original vs. revised diagnostic criteria for LC-MGUS

Light Chain MGUS diagnostic criteria								
	Original	Revised		Original	Revised		Original	Revised
	Abnormal FLC-ratio			Increased **κ**-FLC (mg/L)			Increased **λ**-FLC (mg/L)	
Normal renal function:								
Age <70 years	<0.26/>1.65	<0.44/>2.16	*and*	>19.4	>39	*or*	>26.3	>36.7
Age ≥70 years	----	<0.46/>2.59		----	>55.8		----	>48.0
Impaired renal function:								
eGFR 45-59mL/min/1.73m^2	<0.37/>3.1	<0.46/>2.62		----	>83.6		----	>65.1
eGFR 30-44mL/min/1.73m^2	----	<0.48/>3.38		----	>103.3		----	>73.2
eGFR <30mL/min/1.73m^2	----	<0.54/>3.30		----	>265.1		----	>150.9

Original criteria based on original FLC reference intervals vs. revised criteria based on FLC reference intervals as defined by the iStopMM study. Abbreviations: LC-MGUS light chain monoclonal gammopathy of undetermined significance, FLC free light chain, eGFR estimated glomerular filtration rate

Supplementary Table 4:
Descriptive data of individuals with LC-MGUS included in the progression analysis

	LC-MGUS revised	LC-MGUS original	Reclassified individuals
**Characteristic**	**N = 191**	**N = 334**	**N = 148**
Age (years), Median (IQR)	71 (64 – 80)	72 (66 – 78)	72 (66 – 77)
Sex, n (%)			
F	72 (38)	138 (41)	67 (45)
M	119 (62)	196 (59)	81 (55)
Lambda (mg/L), Median (IQR)	27 (13-139)	18 (12-44)	17 (12-23)
Kappa (mg/L), Median (IQR)	86 (25-207)	46 (27-131)	35 (27 – 47)
FLC-ratio*, Median (IQR)	9.66 (5.33-19.53)	4.26 (2.21-11.83)	2.16 (1.89 – 2.57)
eGFR, n (%)			
>=60	148 (77)	274 (82)	130 (88)
<60	43 (23)	60 (18)	18 (12)
Lambda LC MGUS, n (%)	69 (36)	67 (20)	2 (1.4)
Kappa LC MGUS, n (%)	122 (64)	267 (80)	146 (98.6)
Immunoparesis in at least 1 Ig, n (%)	68 (36)	87 (26)	20 (14)
Immunoparesis in >1 Ig, n (%)	20 (11)	22 (6.6)	2 (1.4)
Events during follow-up, n (%)			
Deaths within follow-up, n (%)	58 (30)	108 (32)	52 (35)
Multiple myeloma, n (%)	11 (5.8)	9 (2.7)	0 (0)
Lymphoma, n (%)	2 (1)	4 (1.2)	2 (1.4)
AL amyloidosis, n (%)	7 (3.7)	6 (1.8)	0 (0)
Morbus Waldenström, n (%)	1 (0.5)	1 (0.3)	0 (0)

*involved/uninvolved FLC-ratio. MGUS: monoclonal gammopathy of undetermined significance, IQR: interquar-tile range, FLC: free light chain, eGFR: estimated glomerular filtration rate, Ig: immunoglobulin, AL amyloid light chain

Supplementary Figure 1: Cumulative incidence curves showing the risk of progression to MM and AL amyloidosis in individuals with LC-MGUS, using Aalen-Johansson estimation accounting for death as competing risk. The three curves represent the revised LC-MGUS group (green), the original LC-MGUS group (red) and the reclassified group (blue). Abbreviations: LC MGUS light chain monoclonal gammopathy of undetermined significance, MM multiple myeloma, AL amyloid light chain

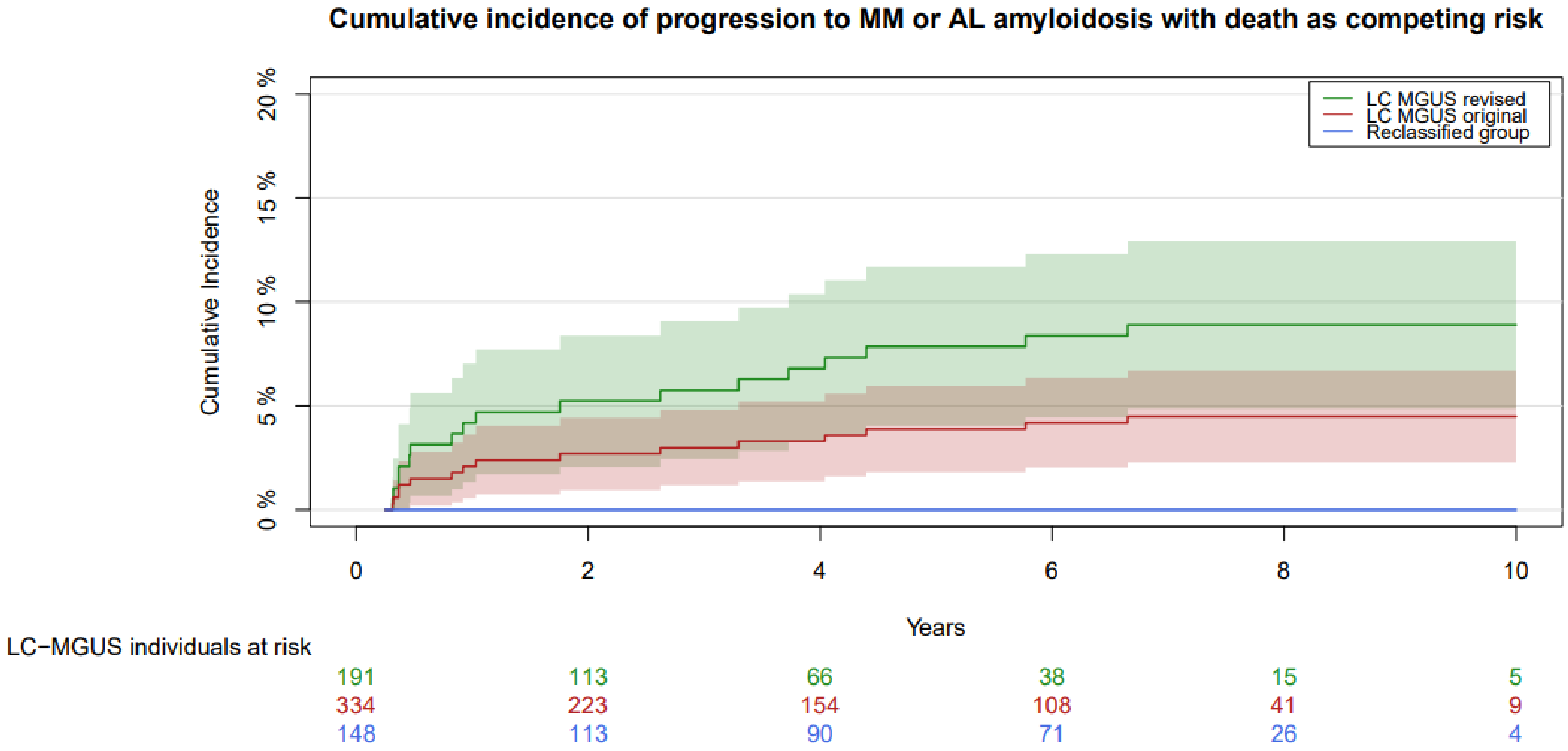

Supplementary Figure 2: Cumulative incidence curves showing the risk of death in individuals with LC-MGUS prior to/without progression to any LPD, using Aalen-Johansson estimation. The three curves represent the revised LC-MGUS group (green), the original LC-MGUS group (red) and the reclassified group (blue). Abbreviations: LC MGUS light chain monoclonal gammopathy of undetermined significance, LPD lymphoproliferative disease

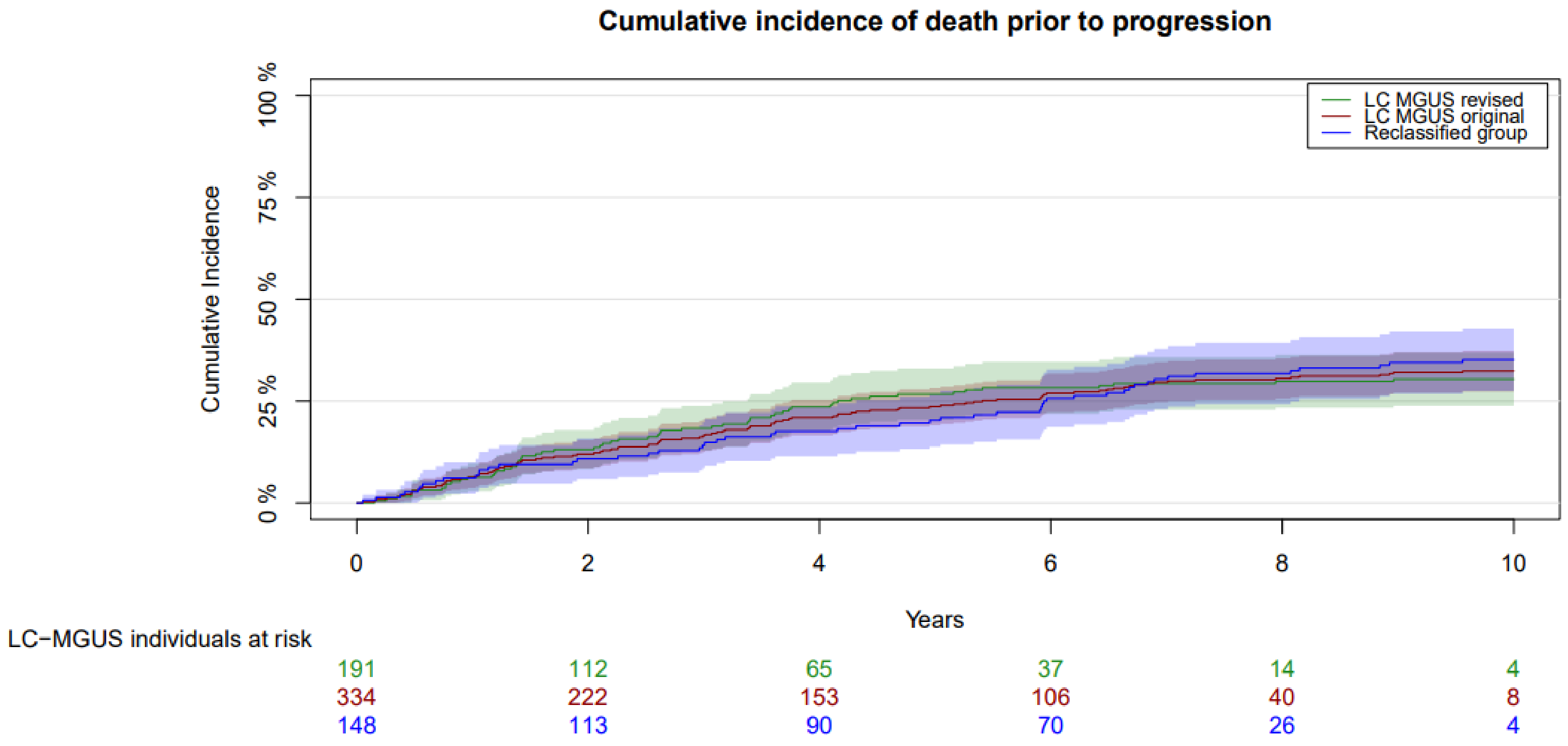

Supplementary Table 5: Risk factors for progression to a lymphoproliferative disorder. The table presents hazard ratios (HRs) for progression according to each risk factor, shown as unadjusted estimates and estimates adjusted for age and sex. Significant results in bold. Abbreviations: HR: Hazard ratio, CI: confidence interval, FLC: free light chain. *Immunoparesis defined as suppression of at least one immunoglobulin.

Risk factor	Crude HR	Adjusted HR
**Lambda isotype**	HR 1.96 (95% CI: 0.81-4.71)	**HR 2.51 (95% CI: 1.01-6.20)**
**Immunoparesis***	HR 0.99 (95% CI: 0.39-2.59)	HR 1.01 (95% CI: 0.37-2.70)
**FLC ratio >8**	HR 0.91 (95% CI: 0.38-2.19)	HR 1.10 (95% CI: 0.44-2.77)
**FLC ratio >10**	HR 0.78 (95% CI: 0.32-1.91)	HR 0.97 (95% CI: 0.38-2.45)
